# Prevalence and Phylogenetic Analysis of Lipoprotein-Gene *ragB-1* of *Porphyromonas gingivalis*—A Pilot Study

**DOI:** 10.3390/antibiotics12091458

**Published:** 2023-09-19

**Authors:** Sarah Böcher, Hendrik L. Meyer, Evdokia Dafni, Georg Conrads

**Affiliations:** 1 Department of Operative Dentistry, Periodontology and Preventive Dentistry, Rheinisch-Westfälische Technische Hochschule (RWTH) University Hospital, Pauwelsstrasse 30, 52074 Aachen, Germany; 2 Division of Oral Microbiology and Immunology, Department of Operative Dentistry, Periodontology and Preventive Dentistry, Rheinisch-Westfälische Technische Hochschule (RWTH) University Hospital, Pauwelsstrasse 30, 52074 Aachen, Germanygconrads@ukaachen.de (G.C.)

**Keywords:** *Porphyromonas gingivalis*, phylogeny, lipoprotein RagB, periodontal diseases, virulence

## Abstract

*Porphyromonas gingivalis* (*P.g.*) is a key pathogen involved in periodontal diseases. The aim of this study was to investigate the prevalence and phylogenetic origin of the lipoprotein-gene *ragB* in its most virulent variant, *ragB-1* (co-transcribed with *ragA-1* as locus *rag-1*), in different *P.g.* strains collected worldwide. A total of 138 *P.g.* strains were analyzed for the presence of *ragB-1* by pooled analysis and subsequently individual PCRs. Sequencing a core fragment of *ragB-1* of the individual strains made it possible to carry out a phylogenetic classification using sequence alignment. In total, 22 of the 138 *P.g.* strains tested positive for *ragB-1*, corresponding to a prevalence of 16%. The fragment investigated was highly conserved, with variations in the base sequence detected in only three strains (OMI 1072, OMI 1081, and OMI 1074). In two strains, namely OMI 1072 (original name: I-433) and OMI 1081 (original name: I-372), which originate from monkeys, two amino-acid alterations were apparent. Since *ragB-1* has also been found in animal strains, it may be concluded that *rag-1* was transferred from animals to humans and that this originally virulent variant was weakened by mutations over time so that new, less virulent, adapted commensal versions of *rag* (*rag-2*, *-3*, *and -4*), with *P.g.* as the host, evolved.

## 1. Introduction

*Porphyromonas gingivalis* (*P. gingivalis*, *P.g.*) is a black-pigmented, immobile, gram-negative, anaerobic rod [1], which is considered a key pathogen (or pathobiont) involved in periodontal diseases. There is considerable heterogeneity between the different isolates, including variable virulence [2]. Molecular typing studies have shown that strains of certain genotypes are more frequently associated with disease (periodontitis) [3,4,5,6,7]. Animal models have also confirmed that some strains are more pathogenic than others. However, definitive factors specific to virulent or avirulent *P.g.* strains have not yet been isolated. Well-known virulence factors do not fully explain the differences in pathogenicity between different strains [2,7,8]. Deeper genetic analysis of *P.g.* is therefore crucial for understanding its properties and the role of its individual virulence factors [9]. A better understanding of the factors that determine the variance of strains, in terms of their pathogenic potential, is, in turn, important for improving diagnostic tools and therapeutic strategies [7].

RagA and RagB proteins are major components of the outer membrane of *P.g.* and have been associated with *P.g.* virulence by contributing to subcutaneous lesion development, epithelial cell invasion, and efficient growth of the pathogen by acting as a transport system for nutrients [10] but have been little studied in comparison with other virulence factors. They are proteins that are 115 (RagA) and 55 kDa (RagB) in size, encoded by genes PG0185 (*ragA*) (3.1 kb) and PG0186 (*ragB*) (1.5 kb) [11,12,13,14,15]. The *ragA* and *ragB* genes are co-transcribed to a single, approximately 4.7 kb, mRNA [16], with *ragB* located immediately (30 bp) downstream of the *ragA* gene [7]. Mutants with deletion of either *ragA* or *ragB* have been shown to be phenotypically negative for both proteins [17]. RagAB occurs as a receptor pair on the bacterial cell surface of *P.g.* and consists of the TonB-dependent-transporter (TBDT) RagA (PG0185) and the surface-lipoprotein RagB (PG0186) [10,18]. The protein pair is also considered to exhibit typical features of a TonB-dependent outer-membrane receptor [13,14].

The X-ray crystal structure of RagAB, purified from *P.g.* W83, revealed a dimeric RagA_2_B_2_ complex with an architecture similar to that of SusCD (starch-utilization system), a TBDT carbohydrate transporter in the outer membrane of *Bacteroides thetaiotaomicron* (*B. thetaiotaomicron*) [7,10,18,19], with RagA showing substantial homology to SusC and RagB to SusD. In this heterotetrameric complex, the single RagA molecule has the structure of a 22-stranded β-barrel with an inserted plug domain, similar to other TBDTs, and is tightly capped on the extracellular side by RagB, which covers a large surface area (approximately 3,850 Å^2^). Full-length RagB contains a signal peptide for export to the outer membrane and a cysteine that is lipidated and anchors the protein to the cell membrane. RagB is a compact molecule of 70 × 55 × 50 Å in size and consists of a single domain centered on a curved helical scaffold. This scaffold consists of ten large α-helices, of which the first eight form four tetratricopeptide repeats (TPRs), each arranged as two helices connected by a linker. Thus, RagB is a tetratricopeptide-repeat protein (TPRP). The TPRs form a right-handed solenoid of helices that is curved and complemented by two capping helices located downstream. The concave surface also bears four large intertwined irregular inserts (A–D) [10,18,20].

**Figure 1:**
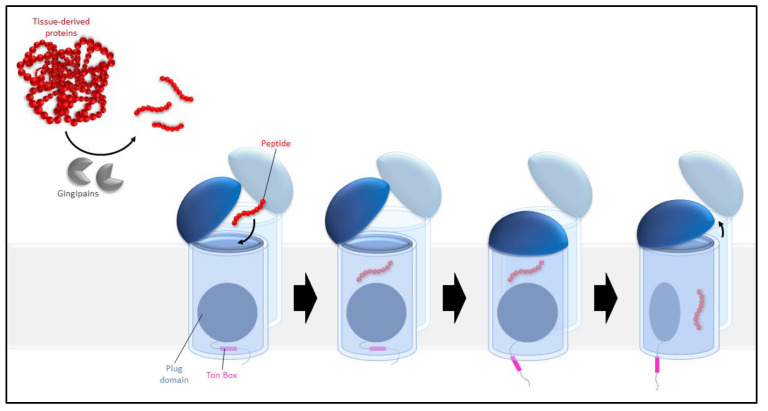
Schematic illustration of the function of RagAB (modified after [18]).

RagAB together form a large, closed internal cavity, containing a molecule of approximately 13 residues long bound to the RagAB interface, which can be considered direct evidence for a “pedal bin” mechanism [18]. The binding of an extracellular substance (peptide) leads to a conformational change that causes disruption of the N-terminal Ton box on the periplasmatic side of the plug domain, making it accessible for interaction with TonB (an inner membrane protein complex). The resulting disruption of the plug domain, in turn, allows the formation of a transport channel to the periplasmatic space that enables translocation of the substrate (Figure 1). Hence, the binding of a peptide to RagAB causes the lid to close and the complex to enter into a transport state. The disrupted state of the Ton box can therefore be considered a signal that ensures that only substrate-loaded transporters form productive complexes with TonB [18].

Substantial structural similarities of RagB with the *B. thetaiotaomicron* SusD and *Tannerella forsythia* (*T. forsythia*, T.f.) NanU (neuraminate uptake system) have already been confirmed. Since the crystal structure of RagB that has been determined shows putative saccharide-binding sites on the molecular surface, as well as bound monosaccharides [10,18,20], it was originally assumed that RagB (together with RagA) is involved in sugar binding and uptake. Although *P.g.* is an asaccharolytic bacterium, this does not mean that it does not require sugar intake. The uptake of saccharides is also necessary for reasons other than energy acquisition—for example, for capsule formation or self-glycosylation of proteins [10,14]. However, analysis of the combined RagAB complex indicated that RagAB is actually a dynamic oligopeptide “acquisition machine” located at the outer membrane, with considerable substrate selectivity, essential for peptide uptake by *P.g.* and effective consumption of proteinaceous nutrients [10,18]. Nagano et al. [12] constructed mutants lacking one—or both—of these two genes (*ragA*, *ragB,* or *ragAB*) and showed that double-deletion mutants lacked both RagA and RagB, while mutants lacking *ragA* showed significantly reduced RagB expression and mutants lacking *ragB* produced only degraded RagA. All mutants grew normally in a nutrient-rich medium and medium containing already digested protein. In a medium containing undigested native protein, growth of the mutants lacking *ragA* and *ragAB* was significantly slower because *P.g.* had to metabolize them first to meet nutritional requirements; however, the amounts and activities of gingipains were unaltered. The authors therefore assumed that mutants lacking RagA and RagB may not be able to take up larger peptides and thus need more time to grow in a medium containing native protein, since smaller peptides must first become available through digestion by the proteases. This confirmed the assumption that cell-surface-associated RagA and RagB are involved in the transport of macromolecules, such as protein-degradation products (peptides), so that strains/mutants lacking these surface molecules grow more slowly due to the lower substrate supply [7,11,21]. This transport is selective, as only certain peptides bind to RagAB and are transported through the outer membrane [18].

RagA, and RagB in particular, are immunodominant antigens [21,22,23]. Early studies have already shown that the IgG response to RagB (determined by serum antibody detection) was higher in adults with periodontitis than in healthy controls [10,21]. More recent studies have also found a strong systemic RagB-recognizing antibody response in individuals with periodontitis [10,22,23]. In vitro studies exposing human inflammatory cells to purified RagB revealed that the expression of several genes encoding pro-inflammatory mediators in monocytes (such as IL-1α, IL-1β, IL-6, and IL-8) was induced in a dose-dependent manner. RagB thus appears to act as a pro-inflammatory mediator. RagB mutants, on the other hand, appear to have a lower inflammatory capacity in comparison with wild-type *P.g.* W83 [10,24]. RagB may therefore play an important role in the etiology of *P.g.*-associated periodontal inflammation [10]. Indeed, expression profiles of RagAB appear to be related to periodontal pocket depth, with increasing expression in deeper pockets [10,14,16], and there seems to be a clear correlation between *rag* gene transcripts in gingival crevicular fluid and clinical indices of periodontitis [10,25].

Sequence analysis of the *ragAB* genes and flanking regions led to the conclusion that the genetic locus was acquired by horizontal gene transfer and that the genetic characteristics of the locus correspond to a pathogenicity island [14]. Thus, the *rag* locus has a low G+C content of 41% in comparison with the G+C content of the whole genome of *P.g.,* which, with its 2,343,479 bp, has an average G+C content of 48.3% [26]. In addition, the locus is flanked by insertion sequences, elements that may have influenced the original acquisition, also indicating a different “foreign” species as the source [14]. The presence of these atypical islands raises the question of how they got there and which unknown microorganism is the “foreign” source of these genes. Studies have shown that more than 40% of the protein sequences encoded in these regions show high homology to proteins of *B. thetaiotaomicron*, an enteric commensal [27,28]. Furthermore, it is conceivable that a gram-negative oral anaerobe acted as a mediator in the transmission [28]. In particular, the close and constant spatial relationships in dental plaque may provide favorable conditions for the transfer of conjugative transposons through cell-to-cell contact [28,29,30]. Thus, the DNA uptake ability has been shown to increase when bacteria are arranged in plaque-like biofilms [28,31,32,33].

However, further research is needed to fully understand the interactions between RagAB and the host immune response [10]. Additionally, a number of studies suggest that the *rag* locus may be a suitable therapeutic target for *P.g.*-associated diseases. Since RagB dominates the *P.g.* antibody response in humans and is an important virulence factor, it has been considered an attractive potential vaccine target. Interestingly, besides RagB, other TonB-dependent outer-membrane receptors have also been investigated as potential vaccine targets for a variety of bacteria [10,34,35,36]. The aim of this study was to determine the prevalence of the virulence-gene PG0186 (lipoprotein-gene *ragB* in its most virulent variant, *ragB-1*) in diverse *Porphyromonas gingivalis* strains collected worldwide. In addition, the exact sequence of a section of the *ragB-1* gene of the different strains was evaluated and a phylogenetic classification made by means of sequence alignment.

## 2. Results

**Figure 2:**
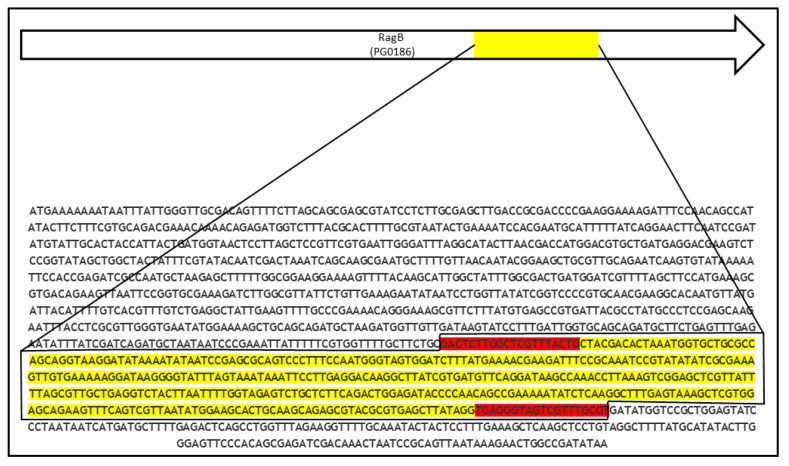
Nucleotide sequence of the *ragB-1* gene of *P. gingivalis* W83 [37]. The part of the *ragB-1* gene sequenced in the present study is highlighted in yellow, and the primer sequences are highlighted in red.

In total, 22 of the 138 *P.g.* strains tested positive, which corresponds to a prevalence of 16%. An overview of all *ragB-1*-positive strains, providing the OMI and other strain numbers used before, as well as their origin (host, year of isolation, and country), is shown in Table 1. The *ragB-1* sequence from OMI 629 (W83) precisely matched the previously published sequence of W83 [37]. However, we found that the primers used did not cover the complete *ragB-1* gene but instead only a section/fragment of 436 bp long (Figure 2), as the complete *ragB-1* gene comprises 1056 bp.

**Figure 3:**
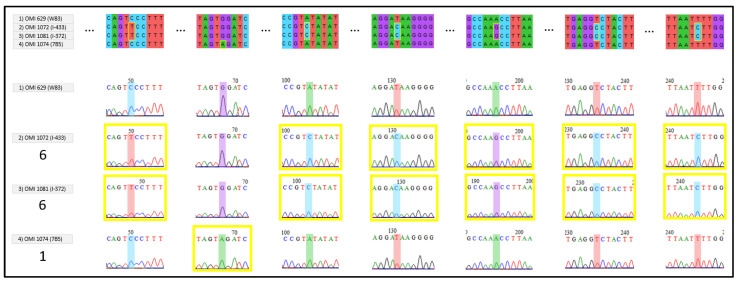
Shown are *ragB-1* variations (outlined in yellow) limited to three *P. gingivalis* strains (OMI 1072, OMI 1081, and OMI 1074). OMI 1072 and OMI 1081 showed the same six base variations at exactly the same positions, while OMI 1074 showed only a single altered base, at a completely different position. Strain OMI 629 (W83) served as a reference.

The sequence of the DNA segment examined in this study proved to be highly conserved. Overall, variations (point mutations, PMs) within the base sequence were only detected in three strains: OMI 1072, OMI 1081 (both six PMs), and OMI 1074 (a single PM). Figure 3 shows the individual base variations with the respective positions of the corresponding chromatograms. OMI 1072 and OMI 1081 showed the same six base variations and at exactly the same positions, while OMI 1074 showed only a single altered base at a completely different position. Strain OMI 629 (W83) served as a reference at the top.

**Figure 4:**
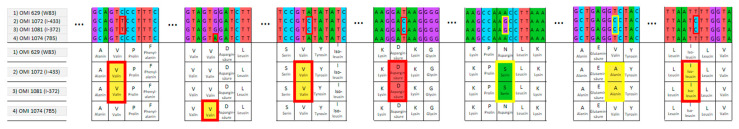
Amino-acid sequences corresponding to alterations found in *ragB-1*. Four of the point mutations detected in OMI 1072 and OMI 1081, as well as the one in OMI 1074, did not cause any change in the resulting amino-acid sequence (synonymous mutations, framed in red). Only two of the point mutations in OMI 1072 and OMI 1081 lead to changes in the translated amino-acid sequence (framed in yellow). Here, alanine was translated instead of valine (both nonpolar/hydrophobic amino acids) and serine was translated instead of asparagine (both polar/neutral amino acids), all conservative (neutral) mutations with no or little effect on RagB functionality.

With regard to the translated amino-acid sequence, four out of six of the PMs detected in OMI 1072 and OMI 1081, as well as the one in OMI 1074, were found to be synonymous mutations, thus with no change in the resulting amino-acid sequence. The remaining two PMs detected in OMI 1072 and OMI 1081 lead, however, to the following changes in the amino-acid sequence: alanine was changed to valine (both nonpolar/hydrophobic amino acids) and serine changed to asparagine (both polar/neutral amino acids). The gene regions affected by the point mutations and the resulting amino-acid sequence are shown in Figure 4.

**Figure 5:**
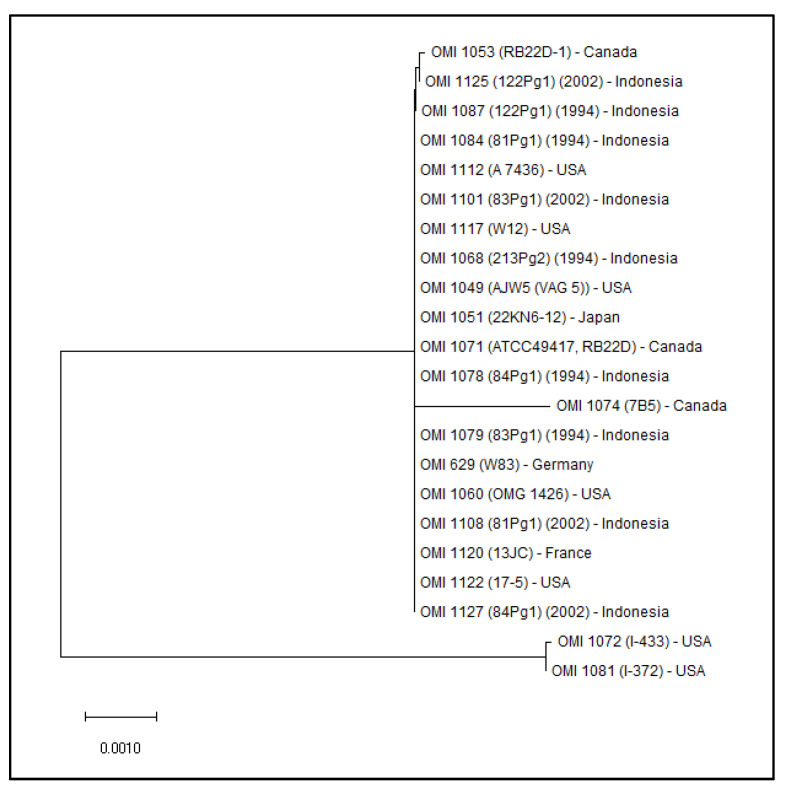
The NJ phylogenetic tree calculated on the basis of 436 bp of *ragB-1* shows identity between all strains, except for OMI 1074 (7B5), OMI 1072 (I-433), and OMI 1081 (I-372), the latter two being *P. gulae* strains from monkeys (program Mega 11, version 11.0.13).

The neighbor-joining (NJ) phylogenetic tree (Figure 5) shows a close relationship among all strains that tested positive for *ragB-1*, except for OMI 1072 (original name: I-433) and OMI 1081 (original name: I-372). These two strains originate from monkeys and were originally classified as *P.g.* However, in 2001, they were re-classified by Fournier et al. [38] as *P. gulae*, which is an animal *P. gingivalis*-like biotype. Interestingly, unlike human *P.g.* strains, these two *P. gulae* strains are catalase-positive [38].

As an expected result of a standard protein BLAST (blastp), the entire sequence of the RagB-1 of *P.g.* W83 showed an analogy to the RagB-/SusD-family nutrient-uptake outer-membrane proteins. It particularly featured a significant analogy to the SusD of *B. thetaiotaomicron*, a TonB-dependent receptor.

Comparison with previously published 3D structural data for RagB-1 [20] shows that the part of RagB-1 investigated in this study (positions 319 to 413) represents part of one of the protein inserts (insert C) and α-helices nos. 12 to 15 [37].

## 3. Materials and Methods

A total of 138 *P.g.* strains available in the strain collection and genome database of the Division of Oral Microbiology and Immunology at RWTH Aachen University Hospital were analyzed for the presence of the lipoprotein-gene *ragB-1* by pooled analysis and later single PCRs, and the results were visualized by gel electrophoresis. Since only a small number of *ragB-1*-positive strains was expected, the 138 *P.g.* strains were first examined in a pooled test procedure, for which the DNA of 10 strains each was pooled. After these pooled PCRs, the strains of each pool that tested positive for *ragB-1* were individually retested for the presence of the *ragB-1* locus. PCRs were performed using a PG0186 primer set (PG0186-F 5′ GACTCTTGCTCGTTTACTG 3′ and PG0186-R 5′ ACGCAAACGACTACCCTCA 3′ [8], synthesized by TIB MOLBIOL Syntheselabor GmbH, Berlin, Germany). After all strains containing a *ragB-1* locus had been identified, the PCR amplicons were purified (NucleoSpin^®^ Gel and PCR Clean-up set, Macherey-Nagel GmbH & Co. KG, Düren, Germany) and sent for DNA sequencing to a service laboratory (LightRun Tube Sequencing Service, Eurofins Genomics Sequencing GmbH, Cologne, Germany). Amplicons were sequenced using the forward primer or both forward and reverse primers and were then assembled into contigs using the Sequence Alignment Editor (BioEdit Sequence Alignment Editor version 7.2.5, Ibis Biosciences, Carlsbad, CA, USA [39,40]). Subsequently, sequence alignment was performed using the MEGA11 program (MEGA11: Molecular Evolutionary Genetics Analysis, version 11 [41]), and a phylogenetic tree (“Construct/Test Neighbor-Joining Tree”) was created from the individual base sequences of all strains that tested positive for the *ragB-1* gene. To evaluate sequence homologies with other known proteins, the standard protein BLAST (blastp) of the National Center for Biotechnology Information (NCBI) was used [37].

## 4. Discussion

In the present study, 22 of 138 *P.g.* strains tested were positive for the presence of *ragB-1*, corresponding to a prevalence of 16%. This is consistent with previous findings that the *rag* locus has four variants and that the most virulent variant, *ragB-1*, is present in only a proportion of strains and clinical isolates [14]. Frandsen et al. [8] reported that the *ragB*(*-1*) locus was found in 13% of strains examined. This percentage is lower than the prevalence of 26% reported for *ragAB-1* in a study by Hall et al. among 168 isolates [7].

However, studies on the prevalence of the *ragB* gene should be interpreted with caution. For example, in the present study, only part of the *ragB(-1)* gene (436 bp) could be examined, as mentioned in the original publication [8]. In turn, different studies have described other primers that were used for the investigation of *ragB* and also examined only part of the *ragB(-1)* gene—which differed, however, from the part examined in the present study. For example, studies by Dolgilevich et al. and Bunte et al. [42,43] used primer pairs that allowed amplification of an upstream part of the *ragB(-1)* gene that was 432 bp long. In fact, the actual *ragB(-1)* gene is 1056 bp long in total, and only a part is covered by established primer pairs [37].

**Figure 6:**
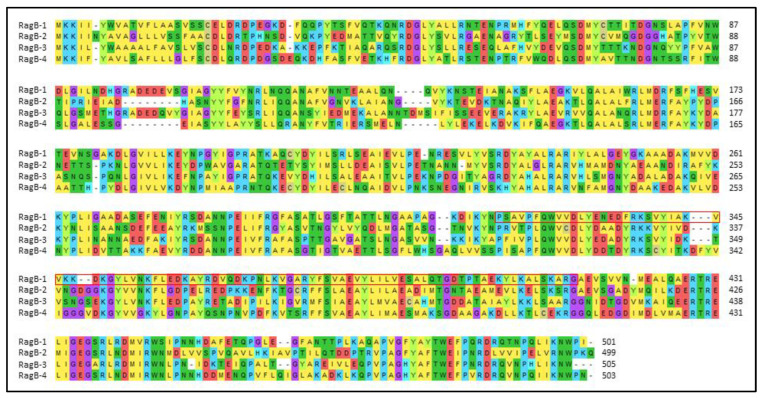
Amino-acid sequences of the different variants of RagB (types 1 to 4), as published by Hall et al., 2005 [7]. The relatively small part of RagB-1 investigated in the present study is outlined in red.

Because prevalence has been investigated on the basis of amplification of different parts of the *ragB* gene, the validity of the results of existing studies on the actual prevalence of the *ragB* gene is limited. Earlier analyses suggest that only a subset of *P.g.* strains contain the *rag* locus [8,14,44], but more-recent whole-genome sequencing clearly shows that the *ragAB* operon is present in probably all strains and clinical isolates analyzed to date. In 2005, Hall et al. [7] demonstrated that there are actually four different variants or alleles of the *rag* locus (*rag-1* to *rag-4*) (Figure 6), with *rag-1* representing the most virulent variant found in W50 and W83, *rag-2* representing the locus with strain A011/9 as a reference, *rag-3* representing the locus of strain QM220 as a reference, and *rag-4* representing the locus of type strain ATCC 33277. This is particularly important, as *rag-2*, *rag-3*, and *rag-4* have frequently been detected in isolates lacking *rag-1* [7,43]. Each of these alleles appears to be common in the population of *P.g.* and is not restricted to a specific geographical region [7]. In a study of 23 *P.g.* strains investigated, *rag-2* and *rag-4* were the most prevalent alleles [45], while Hall et al. [7] found that *rag-1* was the second most common allele among 168 clinical isolates. In total, 26% of these were carriers of *rag-1*, 36% of *rag-2*, 25% of *rag-3*, and 14% of *rag-4*. The presence of different alleles of the *rag* locus also leads to the question of whether certain alleles are associated with different clinical manifestations. In particular, *rag-1* appears to be associated with deep periodontal pockets. Strains shown to be more virulent in mouse models have also been shown to more likely carry *rag-1* than other alleles [7,46]. In another study, *rag-3* and *rag-4* were the predominant genotypes in patients with orthodontic gingivitis and mild-to-moderate forms of periodontitis [25]. The different variants of *ragB* share only 43–56% of amino acids, which explains why the primers used are i) allele-specific and ii) designed on the most-conserved internal sequences, leading to only partial gene amplification [7]. In a previously published study, it was reported that primer efficiency across all gene polymorphisms of *ragAB* for clinical isolates may be impaired [43]. Considering that there are four different variants of *rag* [7], it is even more difficult to draw conclusions about the overall prevalence of *ragB* or different alleles from the currently available data.

In view of the highly variable pathogenicity of *P. gingivalis* in general and *ragB* in particular, studies on the prevalence of the most virulent variant *ragB-1* are particularly important. The question remains of what function the conserved RagB-1 protein fragment has. In comparison with previously published 3D structural data for RagB [20,37], the sequence amplified in this study (positions 319 to 413) appears to represent part of one of the protein inserts (insert C) and α-helices nos. 12 to 15 of RagB-1.

Whether different RagB sequence variants contribute to functional variation remains to be investigated. A new hypothesis based on studies by Madej et al. [18] suggests that different RagABs enable *P.g.* strains to feed on different peptides produced during the degradation of host proteins [10,18]. The authors showed that the ligand-binding site of RagB-1 has an acidic character, which suggests that W83 preferentially takes up basic peptides. Interestingly, this acidic loop (_99_DEDE_102_) is absent in several RagB orthologs, including Rag-2 and Rag-4 [18], but is also found in a truncated form (_99_DED_101_) in RagB-3. Studies have shown that the growth of strains with different RagAB variants is identical on rich medium, whereas on minimal medium with bovine serum albumin (BSA) as the sole carbon source, robust growth was only observed for W83 (RagAB-1), whereas ATCC 33277 (RagAB-4) only grew slowly in contrast [7,18]. In the study by Madej et al. [18], an ATCC 33277 strain was constructed in which *ragAB-4* (or solely *ragB-4)* was replaced by *ragAB-1* (or solely *ragB-1)* from W83. Remarkably, replacement resulted in robust growth of ATCC 33277 on BSA. These results are significant: First, they confirm that the RagAB type does influence the growth of *P. gingivalis* on extracellular protein-derived oligopeptides. They suggest, secondly, that different RagB lipoproteins can form functional complexes with the same RagA transporter and, thirdly, that RagB appears to determine the substrate specificity of the complex.

In addition, the question remains open of where exactly the substrate receptor of the RagAB complex is located. The fact that studies have shown that one and the same RagA protein can bind and transport different substrates by recombination with different RagB “lids” [18] suggests that the substrate specificity of the RagAB complex is determined by RagB. It therefore seems plausible that the “receptor” for this transport is also encoded by the *ragB* gene. Goulas et al. [20] reported that, in most TPRPs, ligand binding occurs at the concave solenoid surface, whereas in *B. thetaiotaomicron,* the *inserts* form the binding site. These inserts differ significantly in sequence and trajectory, which contributes to *B. thetaiotaomicron* having a rather flexible binding site that facilitates binding to oligosaccharide molecules by recognizing the overall 3D shape, rather than the exact composition of the individual monosaccharides [20,47,48].

Because of the sequence homology to SusD of *B. thetaiotaomicron*, which is involved in saccharide uptake, and because RagB of *P. gingivalis* has putative saccharide-binding sites as well, it was originally assumed that its function included saccharide transport [10,14]. However, large differences in inserts A, B, and C indicate that the glycan-binding site of SusD is missing in RagB [20]. Goulas et al. [20] also draw parallels to NanU, where, due to significant differences in the regions shaping the sugar-binding site in SusD, the sialic acid-binding site is still unknown [20,49]. Since studies have now shown that RagAB is indeed a transport system for proteinaceous nutrients [18], the question remains as to why these saccharide-binding sites and bound monosaccharides are present on the RagB protein. Originally, this protein may have served as a saccharide transporter and, after transfer to *P.g.,* gradually converted into a protein transporter, with the saccharide-binding sites remaining as a “remnant” on the surface. Of course, the process could also have taken place in reverse, with the protein complex, originally used as a peptide transporter, being gradually converted into a saccharide transporter after acquisition by *B. thetaiotaomicron*. In conclusion here, however, the question of the origin or remaining function of the saccharide-binding sites and the bound monosaccharides on the surface of RagB remains unanswered.

The genes of the *rag* locus are part of the core genome of *P.g.* but have been reported to be highly variable between strains [43,50]. Nevertheless, the part of the *ragB-1* gene examined in this study was found to be remarkably conserved, and if mutations occurred, they were either synonymous (possibly silent) or at least neutral. On the basis of this high conservation of the section of the *ragB-1* gene examined, it can be assumed to be particularly important. The only two strains with non-synonymous mutations, originally classified as *P.g.,* were identified as *P. gulae* by Fournier et al. in 2001 [38] and are isolates from monkeys. Thus, *P. gulae* is an animal biotype of *P. gingivalis* and—unlike human *P.g.* strains—these two strains are catalase-positive.

Since the virulent *ragB-1* type is also found in animals, it can be speculated i) that *ragAB-1* was probably transferred from animals to humans, ii) that this probably occurred by a gum infection, and iii) that the virulent variant was weakened (tamed) by mutations over time so that new, less virulent, adapted commensal versions of *ragAB,* and thus *P.g.,* have evolved. Further studies are needed in order to elucidate the origin and significance of the *ragAB* locus and its variants, potentially useful as a diagnostic or prognostic marker for *P.g.*-associated periodontitis, and to establish a correlation between structural and functional differences.

## 5. Conclusions

A total of 22 of the 138 *P.g.* strains investigated in this study tested positive for a 436 bp conserved virulence-associated fragment of *ragB-1*, which corresponds to a prevalence of 16%. Variations in the base sequence were only detected in three strains (OMI 1072, OMI 1081, and OMI 1074). In addition, these variations were either synonymous or neutral. From this high conservation, it can be assumed that this section of the *ragB-1* gene must code for an essential function. Since the virulent *ragB-1* type is also found in animals, we speculate that *ragAB-1* was probably transferred from *P. gulae* animal strains to *P. gingivalis* human strains by horizontal gene transfer (as similarly demonstrated for the *fimA* locus encoding *P. gingivalis* long fimbrial stalk protein [51]) and that this originally virulent variant became less virulent by mutations over time as a prerequisite for commensalistic coexistence.

## Figures and Tables

**Table 1:** Overview of all *P. gingivalis*/*P. gulae* strains (22 out of 138, 16%) that tested positive for the *ragB-1* gene.

Strain (OMI)	Species	Original Code	Origin Species	Isolation Year	Country
629	*P. gingivalis*	W83	Human	1991	Bonn, Germany
1049	*P. gingivalis*	AJW5 (VAG 5)	Human	1991	Buffalo, NY, USA
1051	*P. gingivalis*	22KN6-12	Human		Tokushima, Japan
1060	*P. gingivalis/gulae*	OMG 1426	Monkey	1989	Florida, USA
1053	*P. gingivalis*	RB22D-1	Human		Quebec, Canada
1068	*P. gingivalis*	213Pg2	Human	1994	Indonesia
1071	*P. gingivalis*	ATCC49417, RB22D	Human	1993	Quebec, Canada
1072	*P. gingivalis/gulae*	I-433	Monkey	1989	Florida, USA
1074	*P. gingivalis*	7B5	Human		Quebec, Canada
1078	*P. gingivalis*	84Pg1-a	Human	1994	Indonesia
1079	*P. gingivalis*	83Pg1-a	Human	1994	Indonesia
1081	*P. gingivalis/gulae*	I-372	Monkey	1989	Florida, USA
1084	*P. gingivalis*	81Pg1-a	Human	1994	Indonesia
1087	*P. gingivalis*	122Pg1-a	Human	1994	Indonesia
1101	*P. gingivalis*	83Pg1-b	Human	2002	Indonesia
1108	*P. gingivalis*	81Pg1-b	Human	2002	Indonesia
1112	*P. gingivalis*	A 7436	Human		Georgia, USA
1117	*P. gingivalis*	W12	Human		Alabama, USA
1120	*P. gingivalis*	13JC	Human		Rennes, France
1122	*P. gingivalis*	17-5	Human		Minneapolis, MN, USA
1125	*P. gingivalis*	122Pg1-b	Human	2002	Indonesia
1127	*P. gingivalis*	84Pg1-b	Human	2002	Indonesia

Four *ragB-1* strains are persisting in patients (81, 83, 84, 122) from 1994 (a) to 2002 (b).

## Data Availability

The data presented in this study are available on request from the corresponding author S.B. and/or senior author G.C.
